# Using a Latent Variable Method to Develop a Composite, Multidimensional Measure of Structural Racism at the City Level

**DOI:** 10.1007/s40615-023-01695-2

**Published:** 2023-06-29

**Authors:** Michael Siegel, Madeline Rieders, Hannah Rieders, Jinan Moumneh, Julia Asfour, Jinseo Oh, Seungjin Oh

**Affiliations:** Department of Public Health and Community Medicine, Tufts University School of Medicine, 636 Harrison Avenue, Boston, MA 02111 USA

**Keywords:** Racial health disparities, Structural racism, African Americans, Empirical measures

## Abstract

**Introduction:**

Although structural racism is strongly related to racial health disparities, we are not aware of any composite, multidimensional measure of structural racism at the city level in the United States. However, many of the policies, programs, and institutions that create and maintain structural racism are located at the city level. To expand upon previous research, this paper uses a novel measure to measure structural racism at the city level for the non-Hispanic Black population.

**Methods:**

We used confirmatory factor analysis to model the latent construct of structural racism for 776 U.S. cities. The model included six indicators across five dimensions: racial segregation, incarceration, educational attainment, employment, and economic status. We generated factor scores that weighted the indicators in order to produce the best model fit. The resulting factor scores represented the level of structural racism in each city. We demonstrated the utility of this measure by demonstrating its strong correlation with Black-White disparities in firearm homicide rates.

**Results:**

There were profound differences in the magnitude of structural racism across cities. There were also striking differences in the magnitude of the racial disparity in firearm homicide across cities. Structural racism was a significant predictor of the magnitude of these racial disparities in firearm homicide. Each one standard deviation increase in the structural racism factor score increased the firearm homicide rate ratio by a factor of approximately 1.2 (95% confidence interval, 1.1-1.3).

**Conclusions:**

These new measures can be utilized by researchers to relate structural racism to racial health disparities at the city level.

## Introduction

### Structural Racism as a Sentinel Indicator in Public Health Surveillance

Public health surveillance is one of the core functions of public health practice [1]. According to Groseclose and Buckeridge, "Public health surveillance contributes data and information to assess and characterize the burden and distribution of adverse health events, prioritize public health actions, monitor the impact of control measures, and identify emerging health conditions that may have a significant impact upon population health [1 , p. 58]." Surveillance in public health has typically focused on measuring disease rates and individual risk factors for those diseases. However, in January 2023, White, Beatty Moody, and Lawrence, three leading female Black health equity scholars, published a game changing paper in which they argued that structural racism should be considered a "sentinel" indicator in public health surveillance [2]. They found that of 125 U.S. public health surveillance and monitoring systems examined, none collected data on structural racism [2], and they concluded that: "integrating racism as a sentinel indicator in public health surveillance and monitoring systems can advance equity-centered public health praxis and antiracist policy development, implementation, and evaluation, and foster greater accountability among public health actors. The absence of racism data precludes the development of data-driven health objectives and hampers targeted evidence-based action to address health inequities [2, pp. S81,S83]."

Not only is there a lack of public health surveillance systems that monitor structural racism, but there is a dearth of structural racism measures that incorporate multiple dimensions of structural racism and their interaction and which could be used by public health agencies for surveillance purposes [3]. As Hardeman et al. recently pointed out: "empirical research has been slow to quantify structural racism and its impact on public health. What isn’t measured cannot be managed, nor can it be valued [3 , p. 180]." A sincere approach to reducing racial health inequities requires not merely noting the role of structural racism but more importantly, measuring and quantifying structural racism so that it can be valued, managed, and ameliorated.

While several recent studies have quantified structural racism—measured across multiple dimensions—at the neighborhood [4, 5], county [6–8], or state [9–14] levels, we are not aware of any published paper that has quantified structural racism at the city level using a single composite measure that encompasses multiple dimensions. However, it is at the city level that many of the institutions and policies that created and perpetuated structural racism are located. For example, zoning policies, restrictive covenant laws, sundown laws, urban renewal policies, low-income housing policies, rent control policies, policing policies, school policies, budget allocations, and many other housing policies are all promulgated at the city level. Thus, it would be helpful to have a composite measure of structural racism at the city level that could be used to assess the degree of structural racism in each city and monitor it over time.

A surveillance measure of structural racism at the city level would have three major benefits. First, it would change the conversation over structural racism from vague generalities into concrete measures, helping public health practitioners and policy makers understand the magnitude of the problem locally, which makes it more real than quoting national statistics. Second, policy makers typically require data before they act, so quantifying structural racism in cities throughout the country would help public health practitioners to promote action to address it locally. Third, if we are serious about confronting structural racism, we must measure it so that we have a baseline value that can be used to monitor progress and evaluate whether specific policies are working or not. Without city-level measures of structural racism, city public health and policy officials will have neither a baseline to monitor over time to assess progress nor follow-up measures to assure that the health equity of disadvantaged racial/ethnic groups is being properly served. Although measuring structural racism across a large number of cities would not identify the policies that would be effective to reduce racial health inequities, it is essential in order to be able to evaluate the impact of city-level policies that are implemented. As White et al. argue: " While data alone will not serve as a panacea for dismantling racism, the omission to explicitly name, measure, collect, and track racism data severely impedes science and precludes translational efforts to achieve health equity [2, p. S83]." The first aim of this paper, then, was to quantify and make available, for the first time, a composite measure of structural racism for a large number of U.S. cities.

### Previous Measures of Structural Racism

Structural racism is the “the totality of ways in which societies foster racial discrimination, through mutually reinforcing inequitable systems (in housing, education, employment, earnings, benefits, credit, media, health care, criminal justice, and so on) that in turn reinforce discriminatory beliefs, values, and distribution of resources, which together affect the risk of adverse health outcomes" [15 , p. 1454]. Early measures of structural racism tended to focus on just one of the dimensions of structural racism, typically residential segregation [16]. More recently, it has been argued that structural racism measures should be multidimensional in order to account for the reinforcing inequitable systems across multiple dimensions articulated by Bailey et al. in their definition of structural racism [4–6]. Chantarat et al. point out that most existing measures of structural racism are unidimensional, "the multidimensional nature of structural racism is not captured by existing measures used by population health scholars to study health inequities" [5 , p. 1].

Over the past several years, advances have been made both in developing multidimensional measures of structural racism and in creating indices for use at the state and county levels. Several research teams have built composite, multidimensional measures of structural racism at the state and county levels by averaging indicators of structural racism across multiple dimensions [9–14]. These approaches are limited by the assumption that all dimensions of structural racism should be weighted equally [7].

Most recently, several research teams have developed a novel approach that measures structural racism as a multidimensional construct using latent variable models [2]. Instead of weighting all indicators and all dimensions equally, a combination of structural equation modeling and confirmatory factor analysis is used to empirically derive weightings based on an evaluation of model fit with the data. As Hardeman et al. explain: “The approaches assume shared variance between structural racism indicators, allowing researchers to estimate an unbiased effect of a multifaceted system of structural racism on health [2 , p.184].”

Dougherty and colleagues at the Johns Hopkins Bloomberg School of Public Health first presented this approach in a 2020 paper that used confirmatory factor analysis to create a multidimensional latent construct for structural racism at the county level [8]. The final model included seven indicators across the five dimensions of incarceration, education, employment, health care, and housing. The authors found that the structural racism factor scores derived from the structural equation model were associated with higher body mass index among Black people and lower body mass index among White people. This approach was next used by Brown and Homan in a 2022 paper that modeled the latent construct of structural racism at the state level using nine indicators across the five dimensions of incarceration, education, economic status, political participation, and residential segregation [12]. The authors reported that higher levels of structural racism factor scores were associated with worse health outcomes among Black people but had no association with health outcomes among White people. The outcomes examined were self-rated health, body mass index, functional limitations, depressive symptomology, and self-reported mental health. Most recently, in a paper published in December 2022, we followed the approach of Dougherty et al. and Brown and Homan, using confirmatory factor analysis to develop a set of structural racism factor scores for 1,181 counties and reported a strong correlation between this structural racism index and the Black-White racial disparity in firearm homicide rates from 1999-2020 among 395 counties with sufficient data to calculate stable firearm rates [7].

To the best of our knowledge, no study has used the latent variable model approach to derive a measure of structural racism at the city level. The second aim of this research, then, was to expand upon existing research by using the confirmatory factor analysis approach to develop a latent construct of structural racism at the city level, identify the indicators that produce the best model fit, and generate structural racism factor scores for a large number of U.S. cities. Thus, to our knowledge, this paper is not only the first to calculate composite, multidimensional city-level structural racism scores but also the first to use latent variable methods to do so.

### Assessing the Utility of the City-Level Structural Racism Measure

The third aim of this paper was to test the utility of the novel city-level structural racism measure by assessing its relationship with Black-White racial disparities in firearm homicide at the city level. We chose firearm homicide because there is an abundance of evidence that structural racism is related to racial disparities in this outcome, with at least 11 papers documenting this relationship: three at the neighborhood level [17–19], one at the city level [20], one at the county level [7], one at the metropolitan statistical area level [21], and five at the state level [13, 14, 22–24]. In particular, we compared the magnitude of the relationship between changes in the structural racism measure and changes in the ratio of Black to White firearm homicide rates observed in this study with that reported in the one previous study that examined this relationship at the city level [20]. In addition to assessing the utility of the city-level structural racism measure, this analysis adds to the existing literature on structural racism and racial disparities in firearm homicide because it is just the second paper to examine this relationship at the city level and the first to use a multidimensional measure of structural racism.

In summary, our two major research questions are:

1. Can a latent variable be constructed to represent the construct of structural racism at the city level using indicators across multiple dimensions while minimizing the impact of measurement error and confirming a good model fit?
2. Do the factor scores resulting from that model predict Black-White racial disparities in firearm homicide rates across cities?

## Methods

### Design Overview

We developed a unifactorial structural equation model to represent the latent construct of structural racism at the city level. We identified 14 possible indicators covering five dimensions: (1) residential segregation; (2) incarceration; (3) employment; (4) economic status/wealth; and (5) education. Each combination of indicators was modeled with the pre-condition that there needed to be at least one indicator for each of the five dimensions. We selected the model which produced the best model fit statistics. Using the weightings produced by the final model, we generated factor scores for each city which act as estimates of the level of structural racism in that city. All data used in constructing the structural racism index were from 2020, except for jailing rates which were obtained from the 2010 Decennial Census. Next, using multiple regression analysis, we investigated the relationship between the structural racism factor scores and the ratio of the non-Hispanic Black firearm homicide rate to non-Hispanic White firearm homicide rate over the period 1999-2020 for each city.

### Structural Racism Measures and Data Sources

We chose the dimensions to measure structural racism based on our earlier work in developing such a measure at the county level [7]. We explored 14 potential indicators across these dimensions (Table 1): (1) residential segregation (the index of dissimilarity, isolation index, entropy index, and Index of Concentration at the Extremes for racialized economic segregation); (2) education (racial differences in the proportion of persons without a college degree and of persons without a high school degree); (3) employment (racial differences in the proportion of persons in service occupations, the proportion of persons in managerial occupations, and the unemployment rate; (4) economic status and wealth (racial differences in the poverty rate, proportion of persons living in rental housing, the labor non-participation rate, and median household income); and (5) incarceration (racial differences in the proportion of people detained or in local jails). To ensure that higher ratios were indicative of a greater degree of structural racism, we used the White to Black ratios for positive outcomes (median income and percentage of workers in managerial occupations) and Black to White ratios for negative outcomes (all other indicators). For example, a ratio of 2.0 for median household income would indicate that the White median household income is twice that of the Black median household income. A ratio of 2.0 for poverty rate would indicate that the Black poverty rate is twice the White poverty rate. The dimensions used here are similar to the dimensions used in previous studies. The reason why not every dimension—such as environmental justice and political representation—is included is that these data are not available at the city level. We included all dimensions for which we were able to identify reliable data. Table 1 defines each indicator and provides the specific data source and years of data used.

**Table 1:** Dimensions, indicators, definitions, and data sources for development of the City Black Structural Racism Index

**Dimension**	**Indicator**	**Definition**	**Data Source**
Incarceration	Detention or Jailing ratio	Ratio of the Black to White local jailing rate. Local jailing rate is the number of persons in local jails or detention centers per 100,000 population.	United States Census Bureau, Decennial Census, 2010
Segregation	Index of Dissimilarity	Represents the percentage of Black people who would have to move in order to achieve an equal distribution of White and Black people across all blocks within a geographic area.	United States Census Bureau, Decennial Census, 2020
	Isolation Index	It can be interpreted as the extent to which Black members of a block are exposed only to one another.	
	Entropy Index	Measures the spatial distribution of Black and White people within a county.	
	Index of Concentration of Extremes (ICE) for Racialized Economic Segregation	Measures the difference between the number of White people living at over 80^th^ percentile of income and the number of Black people living at below the 20^th^ percentile of income in 2020.	
Economic Status/Wealth	Income ratio	Ratio of median household income for the White population to median household income for the Black population.	United States Census Bureau, American Community Survey’s 5-year estimates for 2020
	Rental ratio	Ratio of the proportion of Black people in rental housing to that of White people in rental housing	
	Poverty ratio	Ratio of the Black poverty rate to the White poverty rate	
	Non-labor participation ratio	Ratio of the Black labor non-participation rate to the White labor non-participation rate. Labor non-participation means not participating in the labor force.	
Education	No high school ratio	Ratio of the proportion of Black people without a high school degree to the proportion of White people without a high school degree	United States Census Bureau, American Community Survey’s 5-year estimates for 2020
	No college ratio	Ratio of the proportion of Black people without a bachelor’s degree to the proportion of White people without a bachelor’s degree	
Employment	Unemployment ratio	Ratio of the Black unemployment rate to the White unemployment rate	United States Census Bureau, American Community Survey’s 5-year estimates for 2020
	Managerial occupation ratio	White to Black ratio of proportion of workers in managerial occupations	
	Service occupation ratio	Black to White ratio of proportion of workers in service occupations	

We used 2020 data for all indicators with the exception of the jailing rates, which were obtained from the 2010 Decennial Census and have not yet been released for 2020. For most indicators, the U.S. Census Bureau does not provide estimates for the non-Hispanic Black population. Therefore, we used data for “Black alone” (defined as anyone of single race who identified as Black). The “White” population consisted of Census estimates of the non-Hispanic White population.

Following Dougherty et al. [8] and Brown and Homan [12] as well as our earlier work at the county level [7], we developed a series of unifactorial, structural equation models containing every combination of indicators but requiring that there be at least one indicator present for each dimension. The models were fit using maximum likelihood estimation with robust standard errors using a standardized set of indicators. We allowed for correlation between error terms for the indicator variables and chose the final covariance structure based on goodness of fit criteria. The four criteria used to assess model fit were: (1) root mean square error of approximation; (2) Tucker-Lewis index; (3) confirmatory fit index; and (4) the standardized root mean square residual. In the factor analyses, we included only counties with a Black population of at least 1,000 to avoid using unstable estimates of parameters for the Black population. The factor scores were standardized so that for any given county, the score represents the number of standard deviations that the county is away from the mean for all counties. All analyses were conducted using the *sem* and *factor* procedures in STATA version 17.

### Structural Racism Final Model

The model with the best fit that emerged from our structural equation modeling and factor analysis contained six indicators and did not include any correlated error terms. The fit statistics for the final model were excellent, with a root mean square error of approximation of 0.045, a confirmatory fit index of 0.967, a Tucker-Lewis fit index of 0.946, and a standardized root mean square residual of 0.032.

**Fig. 1:**
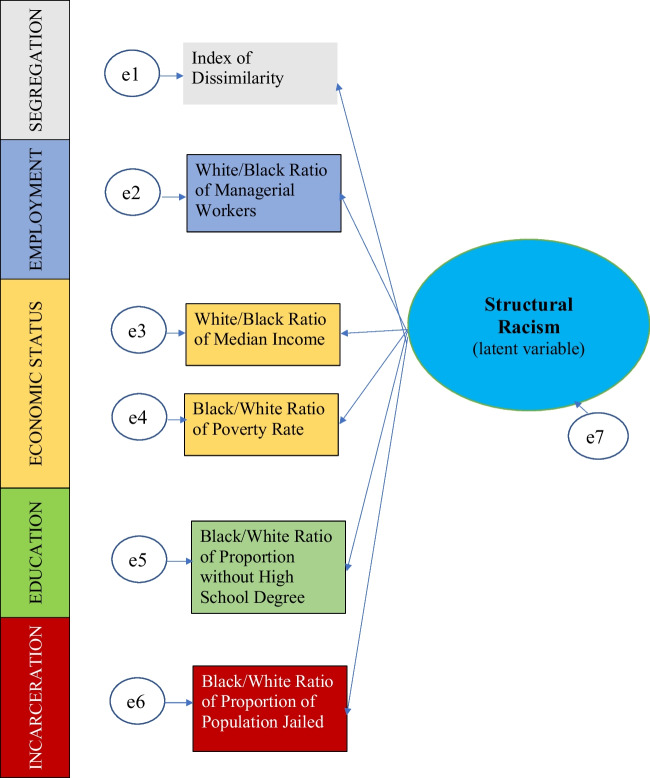
Diagram of final structural equation model. Oval shape indicates the latent variable of structural racism. Rectangular boxes represent the exogenous variables, in this case, indicators covering each of the five dimensions of structural racism considered in this model.

The structural equation model is shown as Figure 1. The indicators in each dimension were as follows:

### Segregation

The segregation dimension included one indicator: the index of dissimilarity, a measure of the differential distribution of two population groups [25]. It was calculated at the block level using data collected from the 2020 Decennial Census and is in a range from 0 to 100, with higher values indicating higher levels of segregation.

### Education

The education dimension included one indicator: the Black to White ratio of the proportion of adults ages 25+ without a high school degree. Data were obtained from the American Community Survey five-year estimates for 2020.

### Employment

The employment dimension included one indicator: the White to Black ratio of the proportion of employees working in managerial occupations. Data were obtained from the American Community Survey five-year estimates for 2020.

### Economic Status/Wealth

The economic status dimension included two indicators: the Black to White ratio of the proportion of the population living in poverty and the White to Black ratio of median household income. Data were obtained from the American Community Survey five-year estimates for 2020.

### Incarceration

The incarceration dimension included one indicator: the Black to White ratio of the local detention or jailing rate in the county. The number of people jailed or detained in a given county was obtained from the 2010 Decennial Census because group quarters data from the 2020 Decennial Census has not yet been released. This indicator could not be calculated for cities that do not have a local jail or detention center, and those cities were not included in the analysis. We were able to calculate jailing/detention ratios for a total of 1,814 cities.

### Structural Racism Factor Scores

We generated standardized factor scores for each city using the weighting of indicators suggested by the final model. These factor scores represent the level of structural racism in each city, expressed in terms of the number of standard deviations its factor score is from the mean for all cities (which was set at zero). Based on data availability, we were able to generate factor scores for 776 cities. These cities comprised a total population of 89.9 million, accounting for 36% of the total U.S. population living in Census-identified cities and comprised 17.5 million non-Hispanic Black people, accounting for 52% of the non-Hispanic Black U.S. population living in Census-identified cities.

### Firearm Homicide Measures and Data Sources

To obtain firearm homicide counts at the city level, we relied upon the FBI’s Uniform Crime Reports (UCR), of which one aspect is the Supplementary Homicide Reports (SHR). Most city police agencies report to this database the number of monthly violent crimes. We included all murders and non-negligent homicides that involved the use of a firearm as being firearm homicides. Information on race/ethnicity and firearm use were missing in a small proportion of cases. Fortunately, Dr. James Fox of Northeastern University has produced multiply-imputed datasets which impute these missing data using five sets of imputed data. Fox kindly provided to us files covering the years 1999-2020 [26]. For each racial/ethnic group (Black non-Hispanic and White non-Hispanic), we tabulated the total number of firearm homicides in each city for which data were reported, summing over the entire period 1999-2020. To generate firearm homicide rates, we divided the total number of homicides by the average race-specific population in each city over the period 1999-2020. We divided the Black firearm homicide rate for each city by the White firearm homicide rate to calculate the racial disparity for that city. Several states did not report data to the SHR for certain years, so we could not use data for cities in those states. We were able to obtain firearm homicide racial disparity data for a total of 467 cities. Of these, 315 cities had non-missing structural racism factor scores. Thus, our analyses relating structural racism and racial disparities in firearm homicide were conducted on a total of 315 cities. These cities accounted for 85% of the non-Hispanic Black population in the 776 cities for which we had structural racism scores.

### Data Analysis

We first log-transformed the homicide rates and rate ratios because of their skewed distribution. Thus, the outcome variables were the logged ratio of the Black to White firearm homicide rate in each city. The main predictor variable was the structural racism factor score for that city. Because we were unable to identify any city-level variables that were correlated with racial disparities in firearm homicide rates and that were not hypothesized to be in the pathway from structural racism to racial disparities in firearm homicide, we conducted bivariate analyses. Because of the logged outcome variable, the exponentiated regression coefficient (multiplied by 100) indicates the percentage change in the outcome variable for each one standard deviation increase in the structural racism factor score.

## Results

**Fig. 2:**
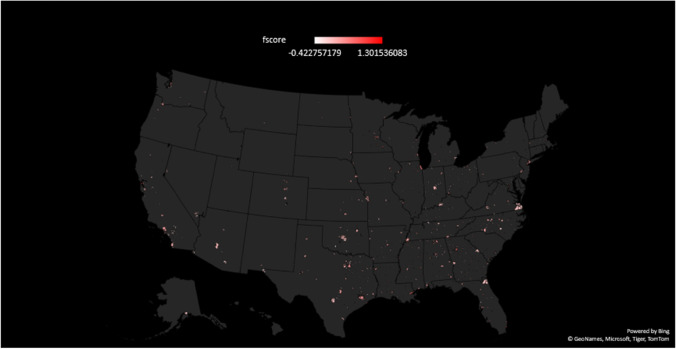
Heat map showing the magnitude of the standardized structural racism factor scores for U.S. cities (N=777). The redder the color, the higher the score. White indicates low levels, pink indicates medium levels, and red indicates high levels of structural racism. The high levels of structural racism in several cities is evidenced by the distinct dark red shading in Atlanta, Miami, Washington, DC, San Francisco, Minneapolis, Chicago, Rochester, Minnesota, Clinton, Iowa and Greenville, South Carolina.

Among the 776 cities, the standardized structural racism factor scores ranged from a low of -2.82 in Stafford Courthouse, Virginia to a high of +6.46 in Washington, Georgia. A color-coded U.S. map displays the standardized structural racism factor scores for each of the 777 cities for which we were able to generate such scores (Figure 2). Table 2 displays the indicators and standardized structural racism factor scores for cities with the 25 highest and 25 lowest scores in the United States. The three cities with the highest standardized structural racism factor scores were Washington, Georgia (+6.46), Sylvester, Georgia (+4.71), and Washington, DC (+4.28). All three had high levels of racial gaps in income (ratios of 2.6 to 3.0), poverty (ratios of 4.0 to 31.0), non-managerial occupations (ratios of 2.0 to 6.3), and proportion of residents without a high school education (ratios of 4.9 to 27.7).

**Table 2:** Summary of city structural racism indicators and standardized factor scores for the top 25 and bottom 25 cities in terms of their factor scores

**City, State**	**Index of Dissimilarity**	**No High School Ratio**	**Managerial Worker Ratio**	**Income Ratio**	**Poverty Ratio**	**Local Jailing Rate Ratio**	**Standardized** **Structural Racism** **Factor Score**
**Top 25 Cities**							
Washington, Georgia	78.31	4.95	2.96	2.85	30.97	2.39	6.46
Sylvester, Georgia	70.60	27.73	6.33	2.64	4.04	0.34	4.71
Washington, DC	69.81	21.91	1.95	3.04	4.34	24.42	4.28
Bamberg, South Carolina	68.10	4.03	2.81	4.26	2.07	2.93	3.56
Miami, Florida	80.26	5.98	2.86	3.56	3.60	4.92	3.49
San Francisco, California	62.06	7.12	1.73	3.89	3.70	15.64	3.45
Macon, Mississippi	59.91	3.25	3.04	3.16	10.24	2.81	3.19
Louisville, Georgia	62.73	3.29	3.41	2.62	13.78	2.32	3.18
Atlanta, Georgia	74.12	8.66	2.12	2.98	3.66	7.53	2.88
Charleston, Missouri	53.89	2.13	3.00	3.78	4.28	0.63	2.78
Minneapolis, Minnesota	67.65	9.69	2.06	2.89	3.35	8.93	2.67
Columbus, Mississippi	63.15	4.91	3.19	3.03	5.58	1.01	2.64
Hernando, Mississippi	52.74	8.11	2.23	2.92	6.27	5.38	2.59
Elizabethtown, North Carolina	57.17	3.18	3.03	3.61	2.99	1.48	2.59
Clayton, Missouri	59.95	16.29	1.18	2.68	1.60	17.54	2.57
Faribault, Minnesota	67.45	7.39	10.58	2.26	4.35	11.63	2.53
Homewood, Alabama	75.26	5.22	1.88	2.88	4.14	13.56	2.50
Rochester, Minnesota	56.12	11.53	1.68	2.39	6.61	6.31	2.42
Amory, Mississippi	67.05	2.03	1.71	3.22	4.34	4.74	2.37
Marianna, Arkansas	57.37	1.76	1.34	3.68	2.55	1.62	2.33
New Orleans, Louisiana	73.45	5.30	2.05	2.84	3.09	4.03	2.27
Kosciusko, Mississippi	57.42	5.30	2.44	3.15	3.20	1.83	2.26
Forest, Mississippi	71.58	1.36	1.98	2.66	7.51	1.41	2.24
Shaker Heights, Ohio	60.60	6.55	1.93	2.87	4.04	5.79	2.23
**Bottom 25 Counties**							
Stafford Courthouse CDP, Virginia	20.92	0.64	0.78	0.48	0.00	1.08	-2.82
Amite City, Louisiana	56.96	0.89	2.87	0.06	0.60	1.35	-2.10
Lynwood, California	52.75	0.81	1.20	0.39	0.24	0.42	-2.02
Jessup CDP, Maryland	35.66	1.16	0.49	0.85	0.00	0.67	-1.98
Franklin, Kentucky	39.28	0.35	2.14	0.59	1.19	4.11	-1.97
Pelham, Alabama	33.06	0.48	1.50	0.97	0.44	9.87	-1.83
Macedonia, Ohio	36.02	0.33	1.38	1.02	0.00	1.98	-1.79
Richmond, Kentucky	32.37	0.85	1.16	0.90	1.45	1.28	-1.78
Elkton, Maryland	28.37	0.62	1.64	1.01	1.43	1.10	-1.75
Acworth, Georgia	33.58	1.11	2.09	0.87	1.05	0.17	-1.75
Adelanto, California	36.37	0.85	1.47	0.81	1.82	0.81	-1.70
Oak Harbor, Washington	37.29	0.80	1.32	0.89	1.10	13.98	-1.70
Bedford Heights, Ohio	42.09	0.71	0.46	0.91	0.83	0.44	-1.68
Tiptonville, Tennessee	26.21	1.26	1.11	1.09	1.48	0.70	-1.68
Culpeper, Virginia	30.83	2.30	1.13	0.93	1.24	3.29	-1.67
Daleville, Alabama	26.71	0.48	0.83	1.32	0.47	2.13	-1.66
Marlboro Village CDP, Maryland	28.59	1.18	1.28	1.20	0.33	1.18	-1.65
Chillicothe, Ohio	41.02	1.40	1.86	0.78	1.02	1.32	-1.65
Warrenton, Virginia	39.29	1.49	2.61	0.84	0.46	3.37	-1.64
Oxford, Alabama	39.21	0.54	0.75	1.07	0.56	2.07	-1.61
Norwalk, California	44.07	1.01	0.81	0.86	0.91	9.91	-1.60
Adamsville, Alabama	45.23	0.53	0.51	0.83	1.68	0.86	-1.58
Rocky Mount, Virginia	40.63	1.34	2.04	0.91	0.69	1.61	-1.55
Hinesville, Georgia	25.31	0.81	0.91	1.27	1.61	2.68	-1.54
Christiansburg, Virginia	38.22	0.92	1.22	1.14	0.13	1.34	-1.53

The three cities with the lowest structural racism factor scores were Stafford Courthouse, Virginia (-2.82), Amite, Louisiana (-2.10), and Lynwood, California (-2.02) (Table 2). All three had less disadvantage among the Black compared to the White population in the areas of poverty, income, and proportion of residents without a high school education.

Among the 110 cities with greater than 25,000 non-Hispanic Black residents (and therefore very stable firearm homicide rate ratio estimates), the magnitude of the Black-White disparity in firearm homicide ranged from a low of 0.80 in Camden, New Jersey to a high of 21.15 in Pittsburgh (Table 3). Following Pittsburgh were Minneapolis, with a rate ratio of 18.38 and San Francisco, with a rate ratio of 17.53. The standardized structural racism factor scores for Pittsburgh, Minneapolis, and San Francisco were +0.89, +2.67, and +3.45, respectively. Only three of the 10 cities with the lowest racial disparities in firearm homicide rates had positive standardized structural racism factor scores, and two of these three were less than 0.4 standard deviations above the mean.

**Table 3:** Magnitude of Black-White racial disparities in firearm homicide rates, 1999-2020, for the top 10 and bottom 10 cities in terms of the level of their disparity, including only cities with a Black population of greater than 25,000 (N=110).

**City, State**	**Ratio of Black to White firearm homicide rate**	**Standardized** **Structural Racism** **Factor Score**
**10 Cities with Highest Racial Disparity**		
Pittsburgh, Pennsylvania	21.15	+0.89
Minneapolis, Minnesota	18.38	+2.67
San Francisco, California	17.53	+3.45
Omaha, Nebraska	14.42	+0.99
Grand Rapids, Michigan	14.36	+0.27
Seattle, Washington	14.34	+1.50
Saint Paul, Minnesota	14.04	+1.43
Boston, Massachusetts	13.38	+1.49
Portland, Oregon	12.00	+0.95
Knoxville, Tennessee	10.37	+0.02
**10 Cities with Lowest Racial Disparity**		
Camden, New Jersey	0.80	-0.69
Hartford, Connecticut	1.39	0.00
San Jose, California	1.41	-0.04
Detroit, Michigan	1.44	-0.25
East Point, Georgia	2.07	-0.20
Houston, Texas	2.10	+1.23
Warner Robins, Georgia	2.13	-1.00
Beaumont, Texas	2.21	+0.20
Waco, Texas	2.23	+0.39
Paterson, New Jersey	2.24	-0.82

**Fig. 3:**
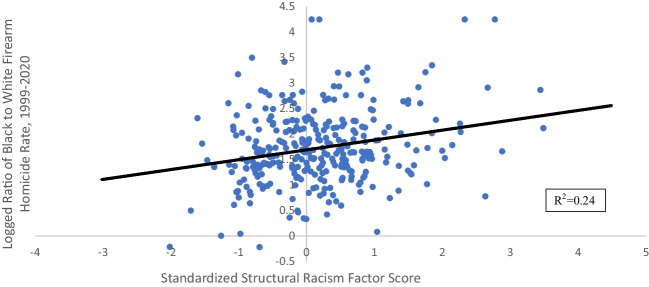
Correlation between the 2020 City Structural Racism Factor Scores and the logged ratio of city-level, 1999-2020 Black to White firearm homicide rates (*N*=315 cities).

At the city level, the correlation between the structural racism factor score and the log of the Black-White racial disparity in firearm homicide was 0.24 (Figure 3), indicating that 6% of the variation in the difference in magnitude in racial disparity in firearm homicide rates across cities was explained by the city structural racism factor score. In a bivariate linear regression analysis, the structural racism factor score was significantly associated with the racial disparity in firearm homicide, with each one standard deviation increase in the factor score increasing the firearm homicide rate ratio by a factor of 1.21 (95% confidence interval, 1.11-1.33) (Table 4).

**Table 4:** Results of linear regression analyses modeling relationship between structural racism factor scores and racial disparity between non-Hispanic Black and non-Hispanic White firearm homicide rates, 1999-2020 (N=315 cities)

**Outcome Variable**	**Regressors**	**Exponentiated Regression Coefficient for Standardized Structural Racism Factor Score**	**95% Confidence Interval**	**p-value**
Log of ratio of Black to White firearm homicide rates	Bivariate	1.21	1.11-1.33	<0.001

The exponentiated regression coefficient (multiplied by 100) indicate the percentage change in the outcome variable for each one standard deviation increase in the structural racism factor score.

## Discussion

To our knowledge, this is the first paper to use confirmatory factor analysis to create a single, composite measure of structural racism at the city level. Previous studies have used this approach to develop structural racism measures at the state [12] and county [7, 8] levels. These studies revealed an association between the structural racism measure and racial disparities in body mass index [8] and firearm homicide [7] at the county level and racial disparities in general physical and mental health at the state level [12]. Like the previous papers, we found a significant association between structural racism factor scores and a major Black-White racial health disparity; in this case, firearm homicide rates. Here, we found that each one standard deviation increase in the structural racism factor score increased the firearm homicide rate ratio by a factor of approximately 1.2. This indicates that over the range of 95% of the distribution of structural racism factor scores, the Black-White racial disparity in firearm homicide differed by approximately two-fold because of the differences in the structural racism measure between cities.

It should be noted that the actual difference in the magnitude of racial disparities in firearm homicide rates across cities was profound. Even restricting the analysis to the 110 cities with a non-Hispanic Black population of greater than 25,000, there was a 21-fold difference in this racial disparity across cities. While Camden, New Jersey experienced approximately a 1:1 ratio of Black to White firearm homicide rates, Pittsburgh experienced a 21:1 ratio. Camden’s standardized structural racism factor score was -0.7, while Pittsburgh’s was +0.9. The magnitude of the racial disparities in firearm homicide at the city level were similar to those observed at the state [14] and county [7] levels.

Our finding that structural racism appears to be associated with greater racial disparities in firearm homicide is consistent with both conflict theory and race stratification theory, which would predict that higher levels of structural racism provide health benefits to White people and health harm to Black people [27–32]. It is for this reason that we chose to model racial disparities in homicide rates rather than absolute race-specific rates. Conflict theory holds that "law and the mechanisms of its enforcement are used by dominant groups in society to minimize threats to their interests posed by those whom they label as dangerous, especially minorities and the poor" [30 , p. 1]. Conflict theory has been invoked as a potential explanation for the racial disparity in firearm homicide, especially in police shootings [30]. Racial stratification theory holds that a system in which race is used as a criterion to assign social positions will result in a disparity in life advantages, including opportunities for employment, education, wealth, and health [31]. According to Finnigan: "Racial stratification increases socioeconomic disadvantage and other risk factors for poor health among minorities relative to Whites" [32 , p. 73]. A key aspect of the theory is that the social forces that create racial stratification result in advantage to the White population at the expense of the Black population. Thus, racial stratification tends not merely to create Black disadvantage, but to create White advantage and therefore profound racial disparities in health.

The magnitude of the observed association between structural racism and the Black-White disparity in firearm homicide rates across cities in this paper is nearly identical to that of the only other paper to examine this relationship at the city level. Wong et al., using the index of dissimilarity as the measure of structural racism, found that each one standard deviation increase in that measure was associated with a 20% increase in the Black-White disparity in firearm homicide rates [20]. Here, we found that each one standard deviation increase in the structural racism factor score was associated with a 20% increase in the Black-White firearm homicide disparity. These results suggest that the novel city-level structural racism index developed here appears to have utility in explaining an important racial health disparity that is known to be associated with structural racism.

Surveillance of structural racism at the city level must become a standard practice of public health practitioners and city policy makers. We hope that the structural racism index created here may be a helpful tool to initiate or expand this critical undertaking. It may also aid public health practitioners in bringing attention to the important role that structural racism plays in creating and maintaining racial health disparities. Ogueji et al. recently reported that U.S. adults have little appreciation for the influence of structural racism on COVID-19 vaccination rates [33]. Providing people with actual numbers to document the magnitude of racial health disparities and the magnitude of structural racism may help the public and policy makers to acknowledge [33] structural racism as a fundamental cause of adverse health.

## Limitations

There are several important limitations of this research. First, since there is no “gold standard” for measuring structural racism, we are unable to confirm that the latent variable we are modeling is a valid measure of structural racism. Second, we were not able to calculate structural racism factor scores for all cities because there are many cities that have a Black population of less than 1,000, which precludes deriving stable estimates. Nevertheless, we were able to measure structural racism in 776 cities, which covers more than half of the non-Hispanic Black U.S. population living in Census-identified cities. Moreover, we were able to include a large number of small and rural cities: of the 776 cities, 364 had a total population of less than 30,000. Third, many cities do not report homicide data to the Supplementary Homicide Report, so the sample size for our analysis of the relationship between structural racism and racial disparities in firearm homicide was limited to 315. Still, these cities comprised 85% of the non-Hispanic Black population of the 776 cities for which we had structural racism scores. Fourth, our structural racism measure does not include several dimensions of structural racism that may be important, such as environmental justice, political participation, credit, and media. Unfortunately, limitations on data at the city level precluded our ability to include these dimensions. Fifth, many of the indicators are based on data sources that could potentially have errors in the classification of race/ethnicity. For example, the race data on those detained or in jail are likely officer-reported rather than self-reported. The likely effect of this misclassification would be to blur the racial distinctions, leading to lower estimates of racial disparities. Finally, we focused on structural racism affecting the non-Hispanic, Black population only. Future research should attempt to extend structural racism measures to other racial/ethnic groups.

## Conclusion

Despite these limitations, this study is the first to derive a multidimensional, composite index to measure structural racism at the city level. This is an important advance because many of the policies, practices, and institutions that create and sustain structural racism reside at the city level. Hopefully, the availability of this new measure will provide public health advocates with a tangible tool to help promote racial justice in our nation’s cities.

## Abbreviations

ACS: American Community Survey
CDC: Centers for Disease Control and Prevention
FBI: Federal Bureau of Investigation
SHR: Supplementary Homicide Report

## References

[1] Groseclose SL, Buckeridge DL (2017). Public health surveillance systems: Recent advances in their use and evaluation. Annu Rev Public Health..

[2] White K, Beatty Moody DL, Lawrence JA (2023). Integrating racism as a sentinel indicator in public health surveillance and monitoring systems. Am J Public Health..

[3] Hardeman R, Homan P, Chantarat T, Davis B, Brown T (2022). Improving the measurement of structural racism to achieve antiracist health policy. Health Aff..

[4] Chantarat T (2019). Structural racism as a system of racial inequities: New approaches and tools.

[5] Chantarat T, Van Riper DC, Hardeman RR (2022). The intricacy of structural racism measurement: A pilot development of a latent-class multidimensional measure. EClinicalMedicine..

[6] Chantarat T, Van Riper DC, Hardeman RR (2022). Multidimensional structural racism predicts birth outcomes for Black and White Minnesotans. Health Serv Res..

[7] Siegel M, Rieders M, Rieders H, Moumneh J, Asfour J, Oh J, Oh S (2022). Measuring structural racism and its association with racial disparities in firearm homicide. J Racial Ethn Health Disparities..

[8] Dougherty GB, Golden SH, Gross AL, Colantuoni E, Dean LT (2020). Measuring structural racism and its association with BMI. Am J Prev Med..

[9] Siegel M, Critchfield-Jain I, Boykin M, Owens A (2021). Actual racial/ethnic disparities in COVID-19 mortality for the non-Hispanic black compared to non-Hispanic white population in 35 US states and their association with structural racism. J Racial Ethn Health Disparities..

[10] Siegel M, Critchfield-Jain I, Boykin M, Owens A, Muratore A, Nunn T, Oh J (2021). Racial/ethnic disparities in state-level COVID-19 vaccination rates and their association with structural racism. J Racial Ethn Health Disparities..

[11] Homan P, Brown TH, King B (2021). Structural intersectionality as a new direction for health disparities research. J Health Soc Behav..

[12] Brown T, Homan P. Structural Racism and Health Stratification in the U.S.: Connecting Theory to Measurement. Published online March 20, 2022. SocArXiv. 10.31235/osf.io/3eacp. . 10.1177/00221465231222924 PMC11110275 38308499

[13] Mesic A, Franklin L, Cansever A, Potter F, Sharma A, Knopov A, Siegel M (2018). The relationship between structural racism and Black-White disparities in fatal police shootings at the state level. J Natl Med Assoc..

[14] Siegel M, Wiklund E (2023). The relationship between state-level structural racism and disparities between the non-Hispanic Black and non-Hispanic White populations in multiple health outcomes. J Natl Med Assoc..

[15] Bailey ZD, Krieger N, Agénor M, Graves J, Kinos N, Bassett MT (2017). Structural racism and health inequities in the USA: evidence and interventions. Lancet..

[16] Riley AR (2018). Neighborhood disadvantage, residential segregation, and beyond—lessons for studying structural racism and health. J Racial and Ethnic Health Disparities..

[17] Benns M, Ruther M, Nash N, Bozeman M, Harbrecht B, Miller K (2020). The impact of historical racism on modern gun violence: Redlining in the city of Louisville. KY. Injury..

[18] Poulson M, Neufeld MY, Dechert T, Allee L, Kenzik KM (2021). Historic redlining, structural racism, and firearm violence: A structural equation modeling approach. Lancet Reg Health..

[19] Jacoby SF, Dong B, Beard JH, Wiebe DJ, Morrison CN (2018). The enduring impact of historical and structural racism on urban violence in Philadelphia. Soc Sci Med..

[20] Wong B, Bernstein S, Jay J, Siegel M (2020). Differences in racial disparities in firearm homicide across cities: The role of racial residential segregation and gaps in structural disadvantage. J Natl Med Assoc..

[21] Houghton A, Jackson-Weaver O, Toraih E (2021). Firearm homicide mortality is influenced by structural racism in US metropolitan areas. J Trauma Acute Care Surg..

[22] Unnever JD, Stults BJ, Messner SF (2021). Structural racism and criminal violence: An analysis of state-level variation in homicide. Race Justice..

[23] Knopov A, Rothman EF, Cronin SW (2019). The role of racial residential segregation in Black-White disparities in firearm homicide at the state level in the United States, 1991-2015. J Natl Med Assoc..

[24] Conrick KM, Adhia A, Ellyson A, Haviland MJ, Lyons VH, Mills B, Rowhani-Rahbar A. Race, structural racism and racial disparities in firearm homicide victimisation. Inj Prev. 2022;ip-2022–044788. 10.1136/ip-2022-044788. Online ahead of print. 10.1136/ip-2022-044788 36564165

[25] Massey DS, Denton NA (1993). American Apartheid: Segregation and the Making of the Underclass.

[26] Fox JA (2022). Multiply-imputed Supplementary Homicide Reports File, 1976-2020.

[27] Carmichael S, Hamilton CV (1967). Black Power: The Politics of Liberation in America.

[28] Du Bois WEB (1899). The Philadelphia Negro: A Social Study.

[29] Feagin JR (2006). Structural Racism: A Theory of Oppression.

[30] Petrocelli M, Piquero AR, Smith MR (2003). Conflict theory and racial profiling: An empirical analysis of police traffic stop data. J Crim Justice..

[31] Ariel B, Tankebe J (2018). Racial stratification and multiple outcomes in police stops and searches. Policing Soc..

[32] Finnegan R (2015). Racial and ethnic stratification in the relationship between homeownership and self-rated health. Soc Sci Med..

[33] Ogueji IA, Ceccaldi BMD, Okoloba MM, Maloba M, Adjumo AO, Ogunsola OO (2022). Black people narrate inequalities in healthcare systems that hinder COVID-19 vaccination: Evidence from the USA and the UK. J Afr Am Stud..

